# Implant-Supported Rehabilitation Following Endodontic Failure in a Patient Classified as American Society of Anesthesiologists (ASA) Physical Status III With Chronic Post-Stroke Hemiplegia: A Multidisciplinary Dental Case Report

**DOI:** 10.7759/cureus.114839

**Published:** 2026-08-20

**Authors:** Nayibe Arenas Perez

**Affiliations:** 1 Dentistry, On the Go Braces, West Palm Beach, USA

**Keywords:** asa iii patient, caregiver-assisted hygiene, cbct diagnosis, dental implant rehabilitation, endodontic failure, hemiplegia, implant-supported crown, ischemic stroke, oral rehabilitation, ridge preservation

## Abstract

Patients with chronic neurological disabilities frequently present significant challenges in dental treatment due to functional limitations, impaired oral hygiene, and concerns regarding medical risk. These challenges often result in delayed care or refusal of treatment, particularly when surgical procedures and implant rehabilitation are required. This report describes the multidisciplinary dental management of a 76-year-old male patient classified as American Society of Anesthesiologists (ASA) Physical Status III with a history of ischemic cerebrovascular accident resulting in chronic right-sided hemiplegia, facial paralysis, wheelchair dependence, and reduced ability to perform oral hygiene independently. The patient presented on an emergency basis with severe pain associated with a previously endodontically treated maxillary right second premolar. Clinical and radiographic evaluation, including cone-beam computed tomography (CBCT), revealed persistent periapical infection consistent with endodontic failure. Given the patient's medical history, a comprehensive hospital-based medical evaluation and neurological clearance were obtained prior to treatment. The patient's poor oral hygiene status was addressed through caregiver-assisted oral hygiene instruction, professional dental cleaning, and infection control measures. Following medical approval, the affected tooth was extracted, and alveolar ridge preservation was performed using particulate bone graft material and a resorbable collagen membrane. After a six-month healing period, implant placement was completed using CBCT-guided treatment planning. Three months later, a definitive screw-retained implant-supported crown was fabricated using a digital workflow and successfully delivered. The patient experienced complete resolution of pain, restoration of masticatory function, and improvement in overall quality of life. This case highlights the importance of multidisciplinary dental management, advanced imaging, medical clearance, and caregiver involvement when treating medically stable ASA III patients with chronic post-stroke disabilities. The outcome observed in this carefully selected patient suggests that implant-supported rehabilitation may be successfully performed when appropriate individualized medical assessment, maintenance, and long-term oral hygiene support are available.

## Introduction

The management of medically compromised patients in dental practice requires careful treatment planning, multidisciplinary collaboration, and individualized risk assessment. Patients with a history of ischemic cerebrovascular accident frequently present with long-term neurological deficits, including hemiplegia, facial paralysis, impaired motor function, and dependence on caregivers for daily activities. These limitations often contribute to poor oral hygiene, an increased risk of dental disease, and difficulty accessing dental care [1-4].

For patients classified as American Society of Anesthesiologists (ASA) Physical Status III, the suitability and safety of dental treatment should be determined through individualized assessment of the patient's specific medical condition, comorbidities, functional status, medications, and procedure-related risks. In patients with a previous stroke and stable residual neurological deficits, medical evaluation and appropriate risk stratification are particularly important before surgical procedures or implant rehabilitation. Despite the possibility of treatment in carefully selected and medically stable patients, practitioners may remain hesitant to perform these procedures because of concerns regarding systemic health, postoperative complications, and long-term maintenance [5-10].

Successful implant therapy depends not only on adequate bone volume and surgical planning but also on the patient's ability to maintain oral hygiene. In patients with significant physical disabilities, caregiver participation may become a critical factor influencing treatment outcomes and long-term implant success. Furthermore, implant rehabilitation in medically compromised and dependent patients requires careful patient selection, maintenance planning, and long-term follow-up [11-20].

This case report describes the multidisciplinary dental management of a 76-year-old male patient with chronic post-stroke hemiplegia and facial paralysis who presented with severe pain resulting from endodontic failure of a previously treated maxillary right second premolar. Following comprehensive cone-beam computed tomography (CBCT)-guided diagnosis, medical and neurological clearance, atraumatic tooth extraction, alveolar ridge preservation, implant placement, prosthetic rehabilitation, and caregiver-assisted oral hygiene maintenance, successful functional rehabilitation and long-term oral health were achieved.

## Case presentation

A 76-year-old male patient, ASA Physical Status III, presented on an emergency basis with severe pain associated with a previously endodontically treated maxillary right second premolar (tooth #4). The patient's medical history was significant for a previous ischemic cerebrovascular accident resulting in chronic right-sided hemiplegia, facial paralysis, wheelchair dependence, and limited ability to perform oral hygiene independently. The patient relied on caregiver assistance for daily activities and oral hygiene maintenance.

According to the patient and caregiver, multiple dental offices had declined treatment because of the patient's complex medical condition and perceived surgical risk. Clinical examination revealed generalized plaque accumulation and poor oral hygiene, which were attributed to the patient's physical limitations. Intraoral evaluation demonstrated tenderness associated with tooth #4 and findings consistent with persistent infection. The remaining dentition was also clinically evaluated as part of the initial assessment. Because the patient presented primarily for emergency management of the symptomatic tooth #4, the current report focuses on the diagnosis and rehabilitation of this site. Management of the patient's generalized oral hygiene needs consisted of professional dental cleaning, oral hygiene instruction, caregiver-assisted plaque control, and recommendations for continued dental maintenance.

**Figure 1:**
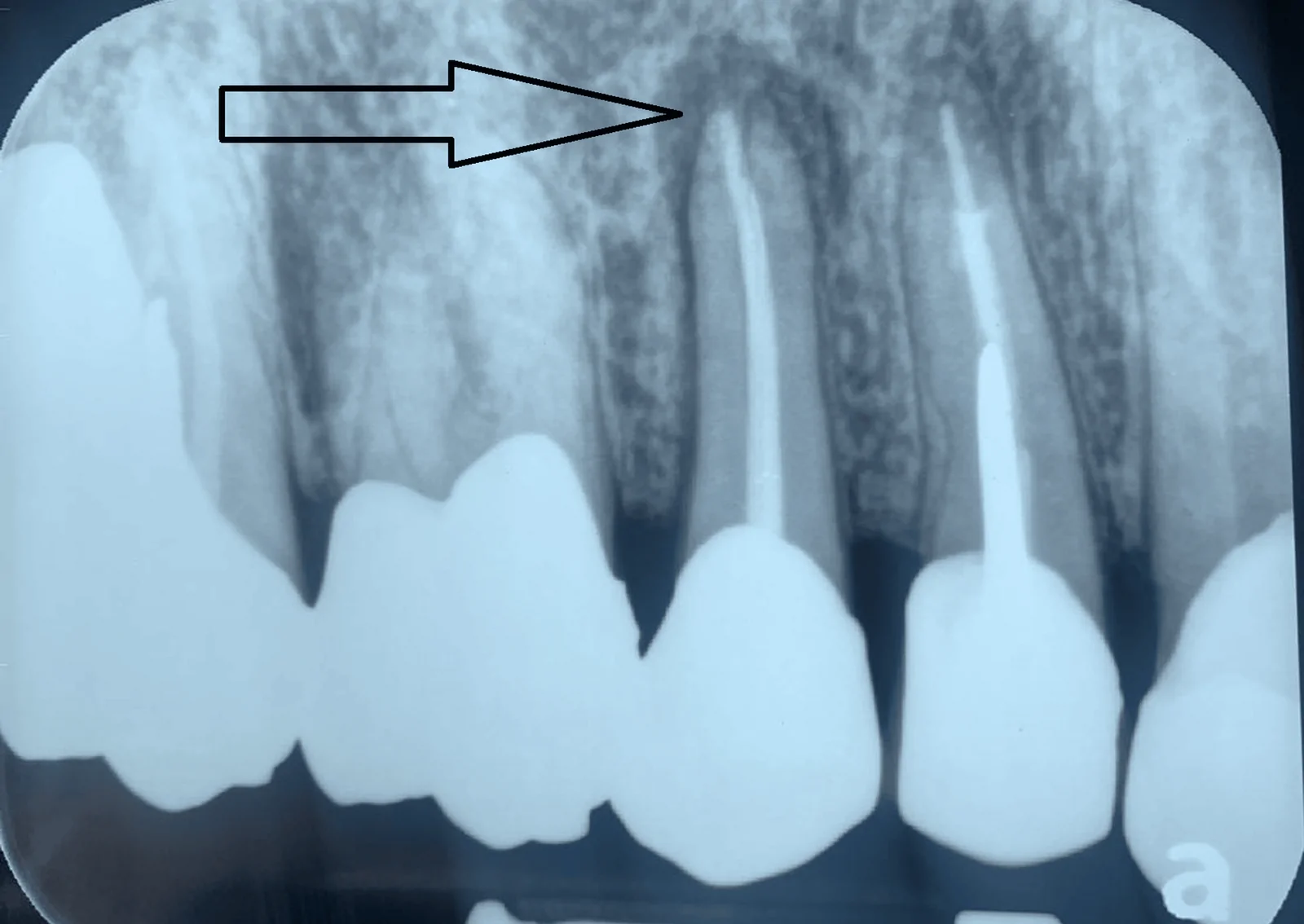
Periapical radiograph demonstrating a persistent periapical radiolucency associated with the endodontically treated maxillary right second premolar (tooth #4). The black arrow indicates the periapical radiolucent lesion, consistent with persistent apical periodontitis secondary to endodontic failure.

Due to the complexity of the case, a comprehensive radiographic evaluation was performed. Periapical radiographs and CBCT were obtained with special precautions because of the patient's wheelchair dependence and limited mobility. The preoperative periapical radiograph demonstrated a persistent periapical radiolucency associated with tooth #4, while CBCT analysis confirmed failure of the previous endodontic treatment and identified the source of the patient's pain (Figure 1).

Given the persistent symptomatic periapical pathology associated with the previously endodontically treated tooth and the planned definitive implant-supported rehabilitation, extraction of tooth #4 was selected after clinical and radiographic evaluation. Endodontic retreatment and apical surgery were considered within the treatment planning process; however, extraction followed by ridge preservation and subsequent implant rehabilitation was selected as the definitive treatment approach for this case.

Given the patient's medical history, he was referred for hospital-based medical evaluation and neurological consultation before surgical treatment. Following a comprehensive individualized assessment, the patient was considered medically stable for dental treatment and received clearance to proceed with the proposed treatment plan.

The patient's oral hygiene status was addressed before surgery through professional dental cleaning, oral hygiene instruction, and caregiver education. Particular emphasis was placed on caregiver-assisted plaque control because long-term treatment success would depend on adequate oral hygiene maintenance. In the setting of persistent symptomatic periapical infection and the planned surgical management, amoxicillin 500 mg three times daily for seven days was prescribed as part of the infection management strategy after consideration of the patient's medical history and planned invasive treatment. Ibuprofen 600 mg was prescribed as needed for pain management.

**Figure 2:**
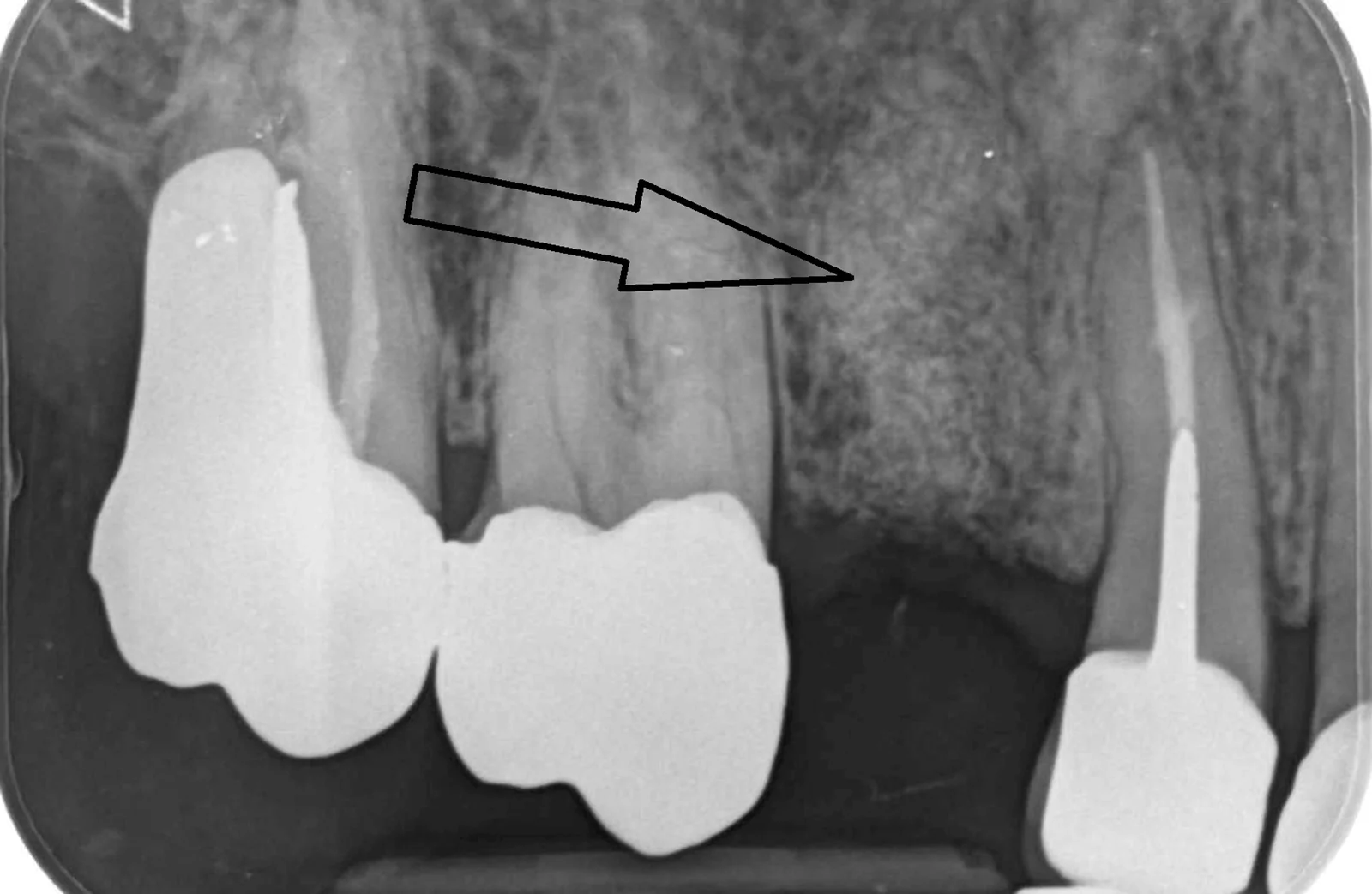
Post-extraction periapical radiograph demonstrating alveolar ridge preservation of the maxillary right second premolar (tooth #4). The black arrow indicates the particulate bone graft filling the extraction socket, which was covered with a resorbable collagen membrane to preserve alveolar ridge dimensions for future implant placement.

Two weeks after completion of the initial preventive phase, tooth #4 was atraumatically extracted under local anesthesia. Following thorough debridement of the extraction socket, alveolar ridge preservation was performed using particulate bone graft material covered with a resorbable collagen membrane (Figure 2). Primary closure was achieved, and postoperative instructions were provided to both the patient and caregiver.

**Figure 3:**
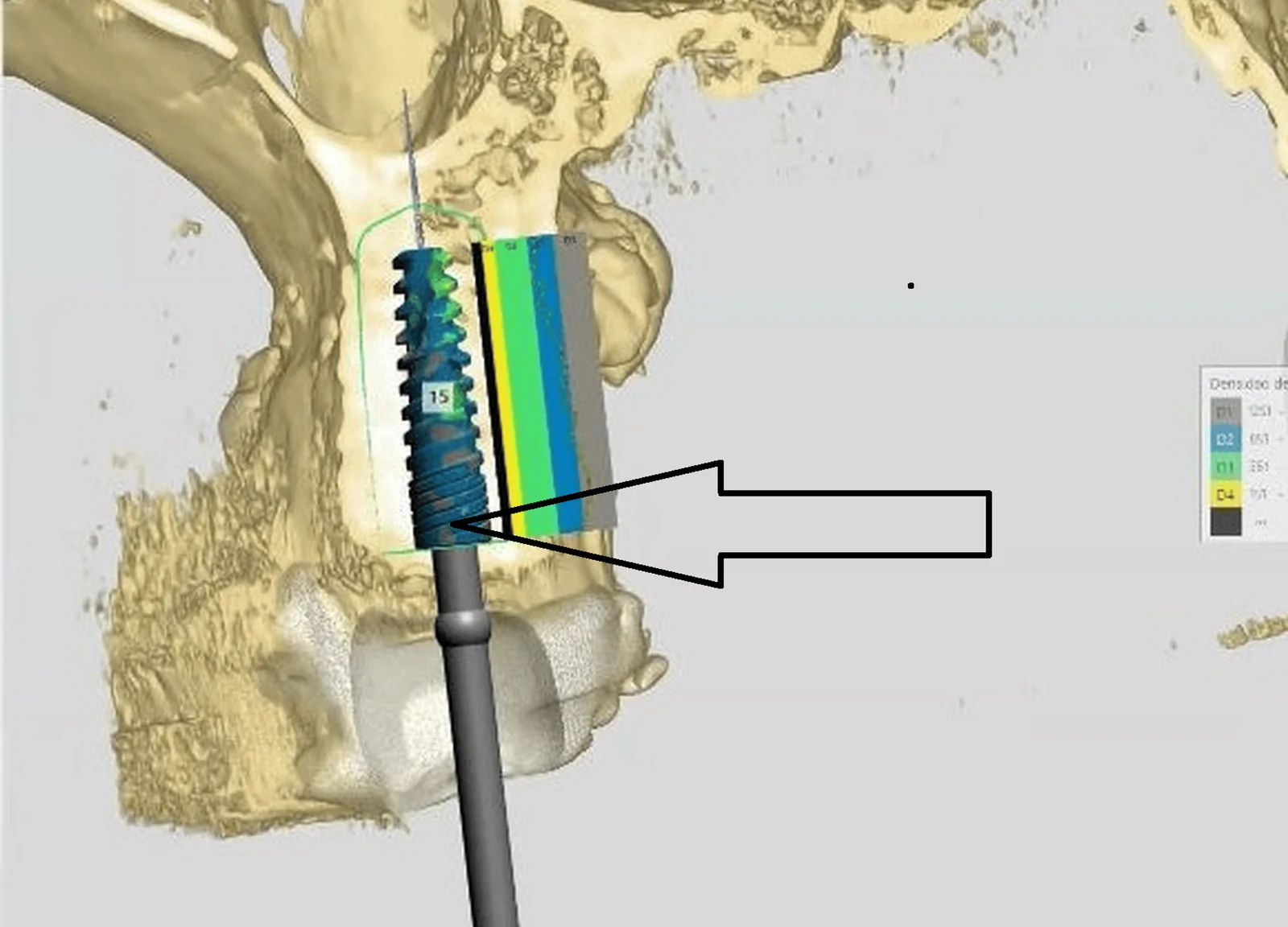
Three-dimensional CBCT-guided digital implant planning for rehabilitation of the maxillary right second premolar (tooth #4). CBCT planning was performed after alveolar ridge preservation to evaluate the regenerated bone and facilitate prosthetically driven implant placement in the maxillary right second premolar (tooth #4) site. The black arrow indicates the virtual implant positioned within the regenerated alveolar ridge, allowing assessment of implant angulation, depth, and surrounding anatomical structures before surgery. CBCT: cone-beam computed tomography

After an uneventful six-month healing period, CBCT evaluation confirmed adequate bone regeneration and preservation of the alveolar ridge. Digital implant planning was subsequently performed using CBCT-guided software to evaluate the regenerated site and determine the ideal implant position, angulation, depth, and relationship to the surrounding anatomical structures before surgery (Figure 3).

**Figure 4:**
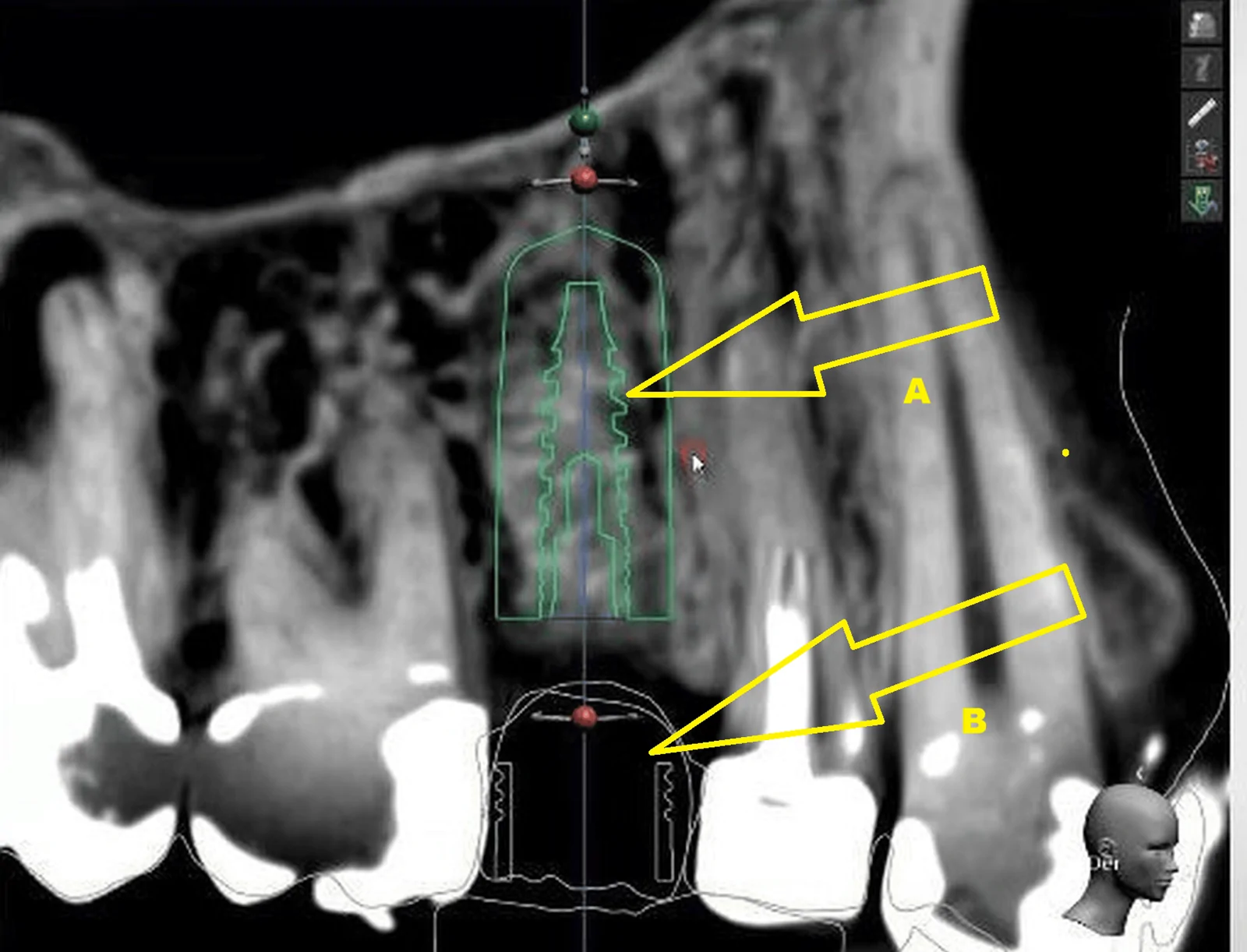
Continued CBCT-guided prosthetically driven implant planning for the maxillary right second premolar site. CBCT-based treatment planning was continued to evaluate the proposed implant position and future prosthetic restoration in this medically complex ASA III patient. Arrow A indicates the virtually planned implant, including its angulation, depth, and relationship to the surrounding anatomical structures. Arrow B indicates the digitally planned crown, which was used to guide prosthetically driven implant positioning and optimize the final restorative outcome. CBCT: cone-beam computed tomography; ASA: American Society of Anesthesiologists

Prosthetically driven planning was then completed by virtually positioning the definitive restoration to optimize implant placement and the anticipated restorative outcome while carefully evaluating the available bone and adjacent anatomical structures (Figure 4).

**Figure 5:**
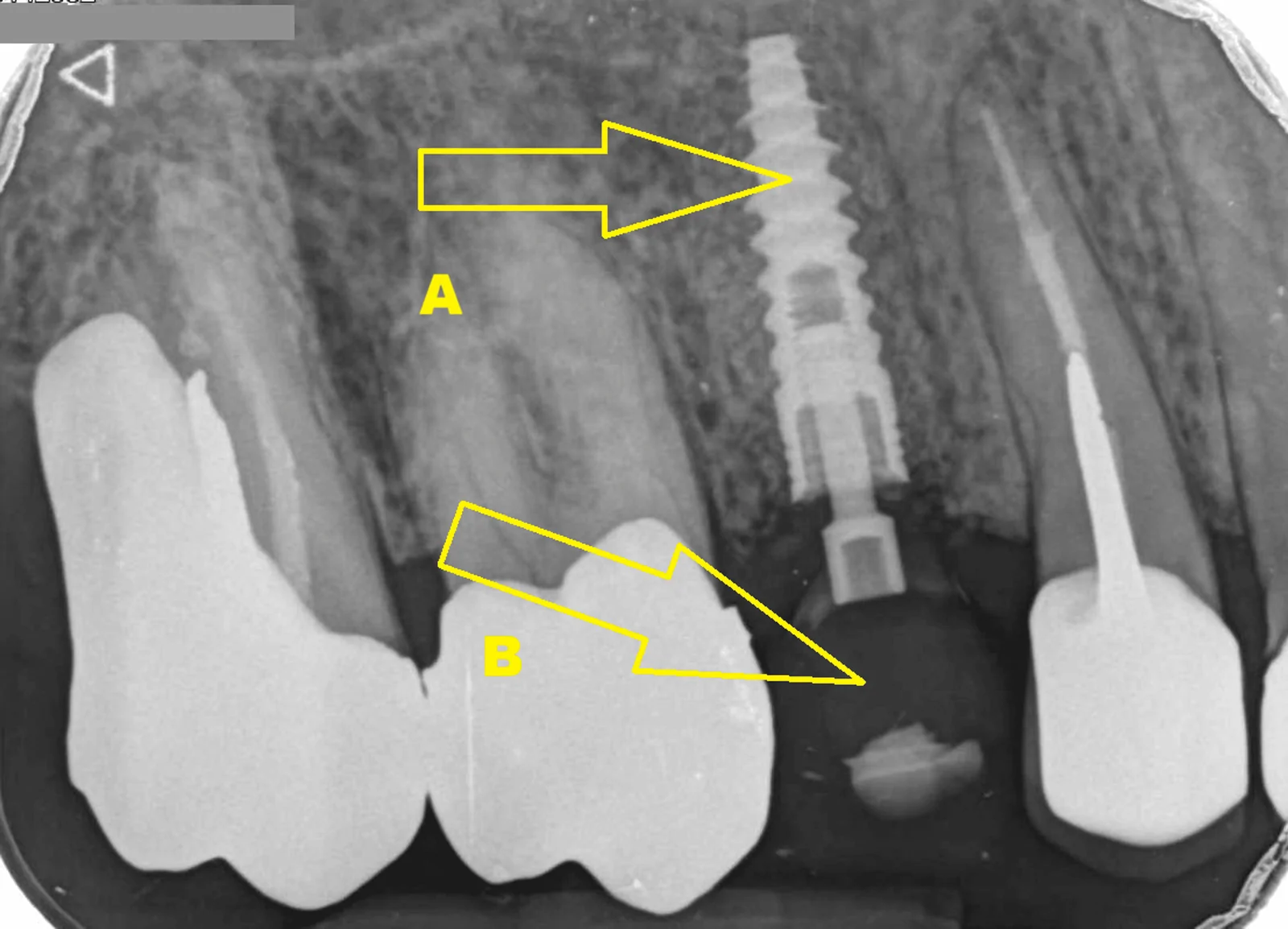
Postoperative radiograph following implant placement and provisional restoration of the maxillary right second premolar (tooth #4). Postoperative periapical radiograph obtained six months after alveolar ridge preservation, demonstrating successful implant placement in the maxillary right second premolar (tooth #4) site. Arrow A indicates the dental implant in its final position. Arrow B indicates the provisional (temporary) crown, which was placed during the provisional restorative phase before fabrication of the definitive implant-supported restoration.

Following completion of the digital planning phase, implant placement was performed according to the CBCT-guided treatment plan. The implant was inserted into the regenerated ridge with satisfactory primary stability, and a provisional restoration was subsequently fabricated during the restorative phase (Figure 5).

**Figure 6:**
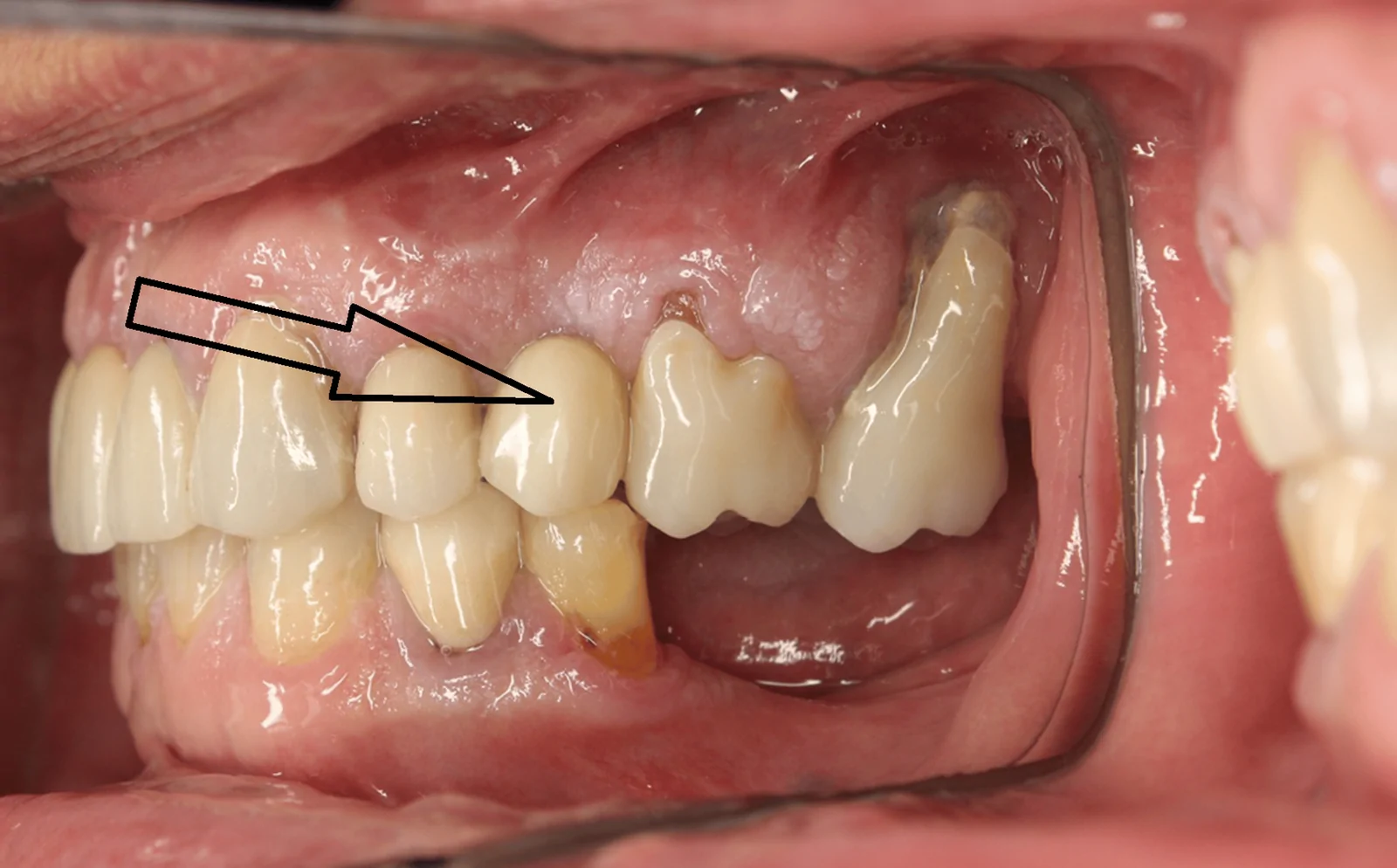
Buccal clinical view of the definitive implant supported crown replacing the maxillary right second premolar (tooth #4). The black arrow indicates the definitive implant supported crown, demonstrating satisfactory esthetic integration with the adjacent dentition. The photograph also shows the patient's compromised posterior dentition, highlighting the importance of long-term maintenance and caregiver-assisted oral hygiene to support the overall prognosis of the implant restoration.

Following an additional three-month osseointegration period, a scan body was connected to the implant to obtain a digital impression of its position. The digital data were transmitted to the dental laboratory for fabrication of a screw-retained implant-supported crown. Two weeks later, the definitive restoration was delivered and successfully inserted. Clinical evaluation from the buccal aspect demonstrated satisfactory esthetic integration with the adjacent dentition (Figure 6).

**Figure 7:**
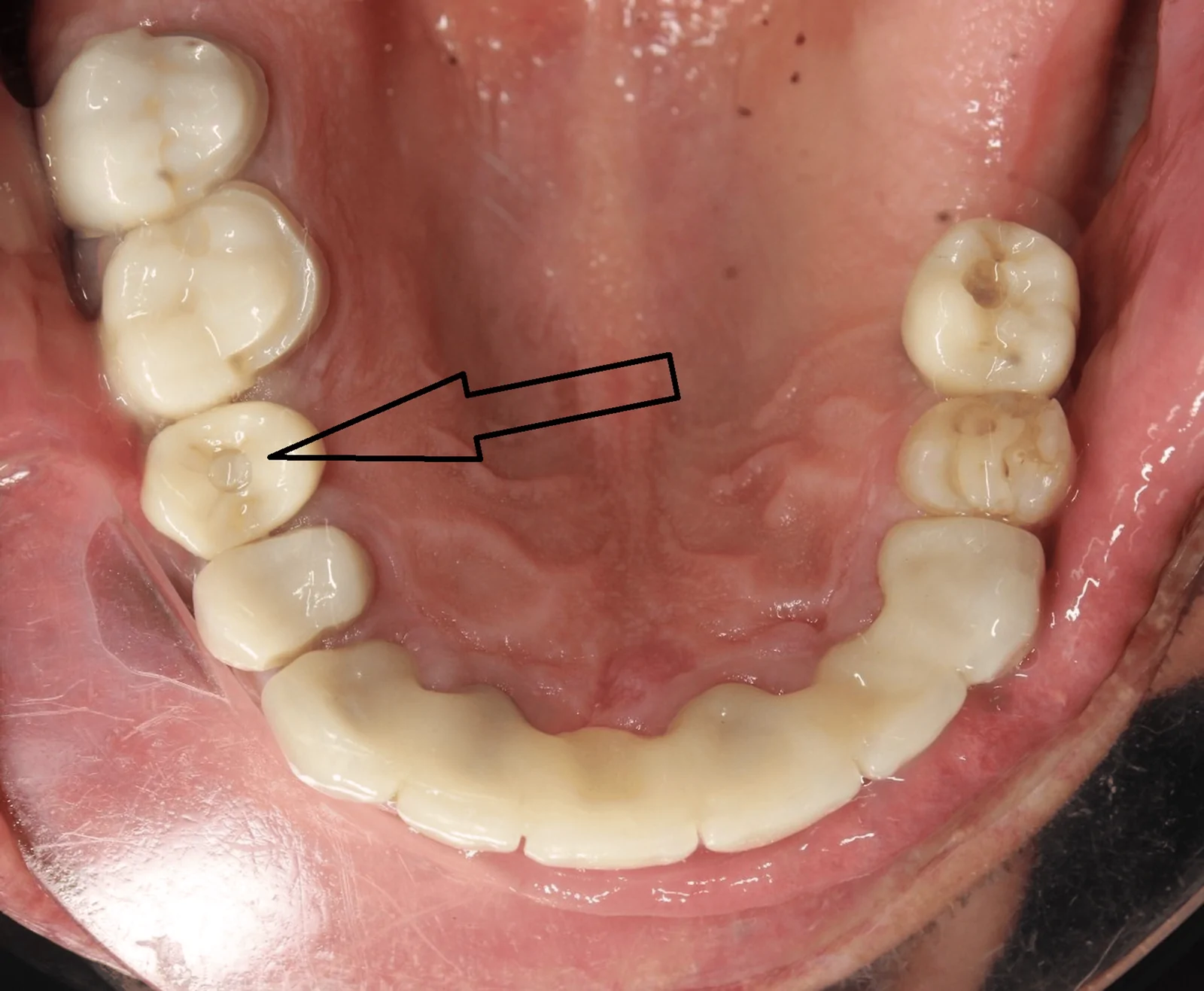
Occlusal view of the definitive implant-supported crown replacing the maxillary right second premolar (tooth #4). The black arrow indicates the definitive implant-supported crown, demonstrating satisfactory occlusal anatomy, contour, and integration with the adjacent dentition.

The definitive crown was clinically evaluated in relation to the opposing and adjacent dentition. Occlusal contacts were assessed and adjusted as necessary before completion of treatment. The available clinical record did not document a specific occlusal guidance classification, such as canine guidance or group function; therefore, a specific occlusal scheme cannot be reliably reported. The occlusal view demonstrated appropriate anatomy, contour, and occlusal relationship of the definitive implant-supported crown (Figure 7).

**Figure 8:**
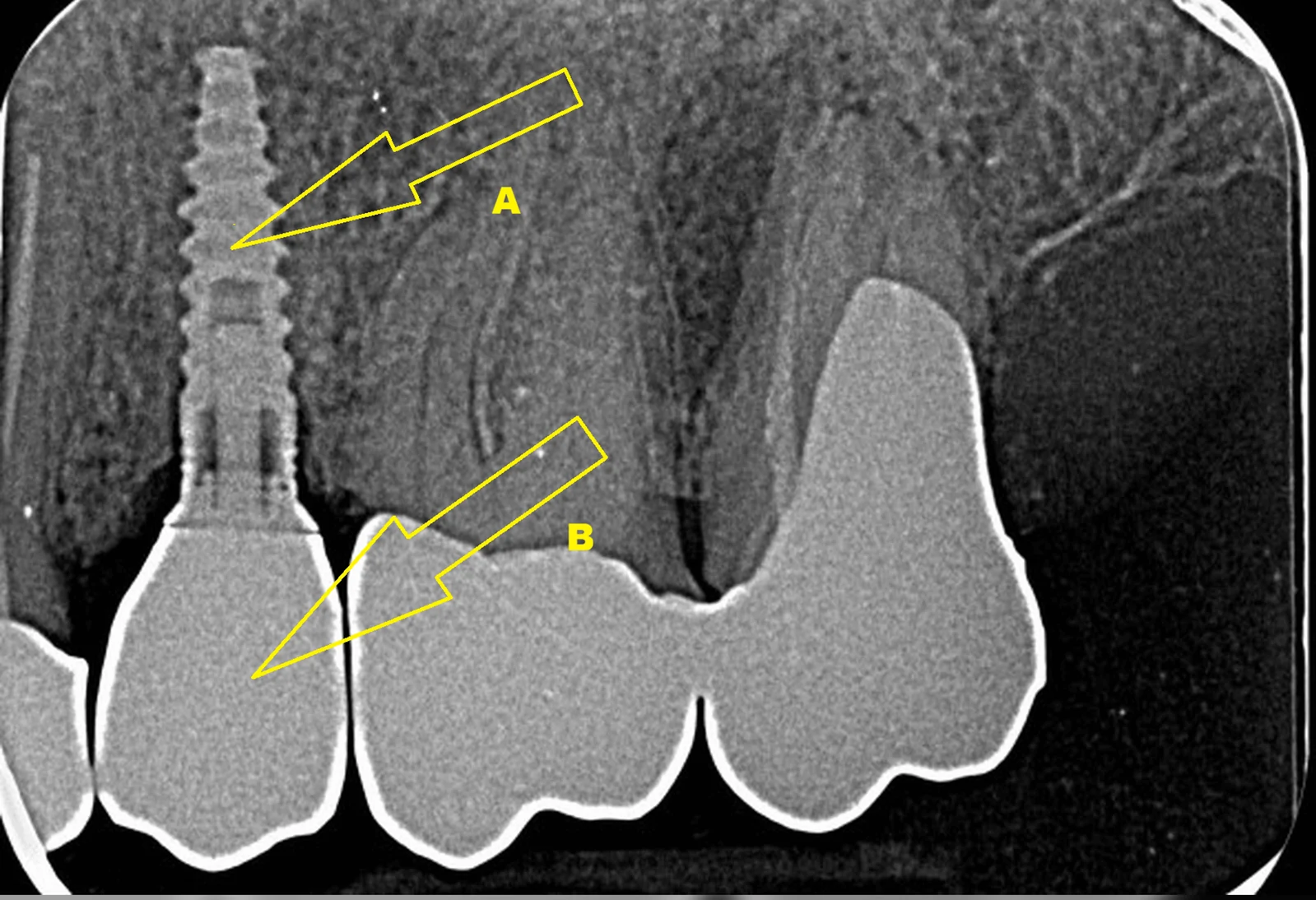
Periapical radiograph demonstrating the definitive implant-supported restoration of the maxillary right second premolar (tooth #4). Postoperative periapical radiograph obtained three months after implant placement, demonstrating successful osseointegration and delivery of the definitive implant-supported restoration replacing the maxillary right second premolar (tooth #4). Arrow A indicates the dental implant supporting the restoration. Arrow B indicates the definitive (permanent) implant-supported crown, demonstrating satisfactory adaptation and functional rehabilitation.

A postoperative periapical radiograph demonstrated satisfactory osseointegration of the implant and appropriate adaptation of the definitive implant-supported crown (Figure 8).

At the follow-up, the patient reported complete resolution of the pain associated with the failed endodontic treatment. Clinical and radiographic examination demonstrated satisfactory healing, absence of clinically detectable implant mobility, and clinically healthy peri-implant tissues. The postoperative radiograph demonstrated maintenance of peri-implant bone support without an evident adverse radiographic finding. Numerical probing depths, bleeding on probing scores, implant stability values, and quantitative marginal bone-level measurements were not recorded in the available clinical documentation; therefore, these objective parameters could not be retrospectively reported and should be considered a limitation when interpreting the treatment outcome. The patient regained functional mastication and reported improvement in overall quality of life. Regular maintenance visits and continued caregiver-assisted oral hygiene were recommended to support long-term treatment maintenance.

## Discussion

The management of patients with significant neurological disabilities presents unique challenges for dental professionals. Individuals with a history of cerebrovascular accident frequently experience impaired motor function, reduced dexterity, facial muscle dysfunction, and dependence on caregivers for activities of daily living. These limitations can negatively affect oral hygiene practices and increase the risk of dental caries, periodontal disease, endodontic complications, and tooth loss [1-4].

The current patient presented with chronic post-stroke hemiplegia, facial paralysis, wheelchair dependence, and poor oral hygiene associated with reduced physical ability. These factors contributed to the complexity of treatment planning and may partially explain why several dental offices declined treatment. However, medical complexity alone should not automatically preclude patients from receiving appropriate dental care when their systemic condition is stable and adequate precautions are taken [5-10].

The successful outcome observed in this case supports the concept that implant-supported rehabilitation can be considered in carefully selected ASA III patients with stable neurological conditions. Treatment decisions should be based on a comprehensive evaluation of medical stability, functional limitations, caregiver support, oral hygiene capability, and patient motivation rather than on the presence of disability alone [11-13].

A critical component of this case was the use of CBCT imaging to identify the source of pain and evaluate the extent of the endodontic failure. Conventional radiographs may provide limited information in complex situations, whereas CBCT allows three-dimensional assessment of periapical pathology, surrounding bone structures, and future implant planning. The information obtained from CBCT contributed significantly to the diagnostic process and the development of a predictable treatment plan [14,15].

Following extraction, alveolar ridge preservation was performed to maintain bone volume and facilitate future implant placement. Ridge preservation procedures have been shown to support implant therapy by reducing post-extraction dimensional changes and improving prosthetically driven treatment planning [16-18].

Oral hygiene maintenance represented a major consideration during treatment planning. Successful implant therapy requires long-term plaque control and regular maintenance. Because the patient was unable to perform adequate oral hygiene independently, caregiver involvement became a determining factor in treatment selection. The caregiver received oral hygiene instructions and assumed an active role in daily plaque control and postoperative care. This support system improved the long-term prognosis of the implant restoration and reduced the likelihood of future peri-implant complications [19].

This case also emphasizes the importance of preserving oral function and quality of life in medically compromised patients. Elimination of chronic infection, relief of pain, restoration of mastication, and improvement in oral health can have substantial benefits for overall well-being. For patients with long-term disabilities, these benefits may contribute significantly to comfort, nutrition, and daily functioning [20].

The present report contributes to the growing body of evidence supporting individualized and multidisciplinary dental management for medically complex patients. Further clinical studies are needed to evaluate long-term implant outcomes in patients with neurological disabilities and to establish evidence-based guidelines for treatment planning and maintenance in this population.

## Conclusions

This case demonstrates that a medically stable ASA III patient with chronic post-stroke neurological deficits may be successfully treated through a multidisciplinary approach that integrates dental care, medical evaluation, neurological consultation, and caregiver support. Careful diagnosis using CBCT, appropriate medical clearance, infection control, alveolar ridge preservation, implant placement, and prosthetic rehabilitation resulted in the successful resolution of pain, restoration of function, and improvement in the patient's quality of life. An essential factor in the success of this treatment was the active involvement of the caregiver, who assumed responsibility for daily oral hygiene maintenance and postoperative care. This support allowed the patient to meet the long-term maintenance requirements necessary for implant therapy despite significant physical limitations.

This report is presented as a contribution to the dental literature and as a practical example for colleagues who may encounter similar patients in clinical practice. Stable ASA III patients with chronic stroke sequelae should not be automatically excluded from advanced dental treatment solely because of their disability. Instead, individualized risk assessment, multidisciplinary collaboration, and adequate caregiver support may facilitate successful treatment outcomes in carefully selected, medically stable patients. This case highlights the importance of a patient-centered, multidisciplinary treatment philosophy and may serve as a practical guide for clinicians seeking to provide comprehensive oral rehabilitation to medically compromised individuals with neurological disabilities.
